# Curculigoside Ameliorates Bone Loss by Influencing Mesenchymal Stem Cell Fate in Aging Mice

**DOI:** 10.3389/fcell.2021.767006

**Published:** 2021-12-03

**Authors:** Na Wang, Ziyi Li, Shilun Li, Yukun Li, Liu Gao, Xiaoxue Bao, Ke Wang, Chang Liu, Peng Xue, Sijing Liu

**Affiliations:** ^1^ Department of Endocrinology, The Third Hospital of Hebei Medical University, Shijiazhuang, China; ^2^ Key Orthopaedic Biomechanics Laboratory of Hebei Province, Shijiazhuang, China; ^3^ Department of Joint Surgery, The Third Hospital of Hebei Medical University, Shijiazhuang, China; ^4^ Editorial Department of Hebei Medical University, Hebei Medical University, Shijiazhuang, China

**Keywords:** osteoporosis, curculigoside, BMSCs, TAZ, MEK-ERK pathway

## Abstract

Senile osteoporosis is characterized by increased bone loss and fat accumulation in marrow. Curculigoside (CCG) is the major bioactive component of Curculigo orchioides, which has been used as anti-osteoporosis therapy for elder patients since antiquity. We aimed to investigate the underlying mechanisms by which CCG regulated the bone-fat balance in marrow of aging mice. In our study, CCG treatment was identified to interfere with the stem cell lineage commitment both *in vivo* and *in vitro*. *In vivo*, CCG promoted the transcriptional co-activator with PDZ-binding motif (TAZ) expression to reverse age-related bone loss and marrow adiposity. *In vitro*, proper concentration of CCG upregulated TAZ expression to increase osteogenesis and decrease adipogenesis of bone marrow mesenchymal stem cells (BMSCs). This regulating effect was discounted by TAZ knockdown or the use of MEK-ERK pathway inhibitor, UO126. Above all, our study confirmed the rescuing effects of CCG on the differential shift from adipogenesis to osteogenesis of BMSCs in aging mice and provided a scientific basis for the clinical use of CCG in senile osteoporosis.

## Introduction

Osteoporosis is increasingly recognized as a major health concern that affects approximately 50% of women and 20% of men over 50 years old (Sambrook and Cooper, 2006). This age-related systemic impairment of bone loss is characterized by decreased osteogenesis and increased adiposity in bone marrow, resulting in more propensity of fragility fracture (Hu et al., 2018; Sanghani-Kerai et al., 2018; Zanker and Duque, 2019). Bone marrow mesenchymal stem cells (BMSCs) could differentiate to osteoblasts or adipocytes. They held a competition relationship during BMSCs’ differentiation (Ambrosi et al., 2017; Muruganandan et al., 2017). The process of stem cell fate determination is constantly changing, and consists of self-renewal and terminal differentiation (Hansen et al., 1999; Dalton, 2015; Urbach and Witte, 2019). Various exogenous and endogenous factors could alter the differentiation switch from adipogenesis to osteogenesis of BMSCs context-dependently (Teitelbaum, 2010; Huang et al., 2018; Wang et al., 2019a). However, the underlying mechanism has not been clearly addressed.

Curculigo orchioides, a small herbal plant belonging to the family Amaryllidacea, has been used as an anti-osteoporosis herb in many Asian countries (Wang et al., 2012; Wu et al., 2012; Tan et al., 2019). The extracts of this plant contain a wide variety of flavonoids, phytosterols, and phenolic compounds, with curculigoside (CCG) identified as the main active substance (Wang et al., 2017). *In vivo* study, CCG was reported to distribute widely in bone marrow and many other tissues after oral administration (Yuan et al., 2015). Cao et al. (2008) discovered that oral administration of CCG prevented bone loss in the tibia of the ovariectomized rats. As was demonstrated, CCG could up-regulate VEGF expression, reverse iron-overload via GPX4, relieve oxidative stress via Akt-FoxO1 axis, and reduce inflammation via NF-κβ signaling transduction to favor the bone remodeling *in vivo* and *in vitro* (Cao et al., 2008; Ma et al., 2011; Tan et al., 2019; Zhang et al., 2019; Wang et al., 2020). In our research, we tried to elucidate if and how CCG switch the differentiation from adipogenesis to osteogenesis of BMSCs in aging mice.

Senile osteoporotic patients often showed an increased adipogenesis in their bone marrow with a reduction of osteoblastogenesis (Rosen et al., 2009). Transcriptional co-activator with PDZ-binding motif (TAZ) has been demonstrated as a nuclear transcription factor playing crucial roles in stem cell differentiation (Kegelman et al., 2018; Wang et al., 2018; Tan and Dai, 2019). As was proved, TAZ combined to runt-related transcription factor 2 (RUNX2) to promote osteogenesis, and interacted with peroxisome proliferator-activated receptorγ (PPARγ) to suppress adipogenesis (Byun et al., 2013; Matsumoto et al., 2016; Wang et al., 2019b). Besides, TAZ has neuro-protection, angiogenesis, and anti-oxidative stress effects, which were important for bone nutrition and regeneration (Lee et al., 2019; Chen et al., 2021; Jeanette et al., 2021). Yu et al. (2018) revealed that PGC-1 alpha targeted TAZ to alter bone-fat balance during skeletal aging. Previously, our study also pointed a molecular link of MEK-ERK pathway to the TAZ during BMSCs osteogenesis (Wang et al., 2018; Wang et al., 2019a). As the MEK-ERK signaling pathway has been intensively investigated in regulating cells differentiation, it exerts our interest on the role of MEK-ERK/TAZ axis in the CCG-mediated differentiation of BMSCs during aging.

Molecular interactions and protein networks of CCG could predict the functions of its compounds as an anti-osteoporotic drug in clinical. However, the underlying mechanisms whereby CCG promotes osteogenesis in bone marrow have not been well established. In this study, we found that CCG induced the switch from adipogenesis to osteogenesis of BMSCs in aging mice and ameliorated the age-related bone loss by influencing the osteoblasts-adipocytes lineage commitment of BMSCs via MEK/ERK-TAZ interaction axis. Altogether, these findings provided a scientific basis for the clinical use of CCG in the geriatric population.

## Materials and Methods

### Mice and *In Vivo* Treatment

Our experiments were approved by the Local Committee of Animal Use and Protection of the Third Hospital of Hebei Medical University. C57BL/6 mice were bred in a room with the environmental temperature ranging from 20 to 24°C and proper humidity ranging from 30 to 70%. The mice had either free access to food or water. The 3-months mice were regarded as the normal control group. In addition, the three groups of 16-months mice would be received vehicle, 50 mg/kg/d or 100 mg/kg/d CCG (Shanghai Standard Biotech, Shanghai, China) by oral administration, respectively, for 2 months. After the 2 months treatment, we considered the above three groups as 18-mon, 50 mg-CCG, and 100 mg-CCG group.

### Micro-Computed Tomography (μCT) Analyses

We fixed the harvested femurs in 4% paraformaldehyde (4°C, 24 h). We used the SkyScan μCT scanner to analyze the bone parameters with 65 kv voltage and 153 μA current. Each image was obtained with high resolution of 9.0 μm per pixel. We used NRecon for image reconstruction (version 1.6), CTAn (version 1.9) for data analysis, and CTVol (version 2.0) for 3D model visualization. With 3D analysis, we collected bone parameters of trabecular bone volume fraction (BV/TV), trabecular bone thickness (Tb.Th), trabecular bone number (Tb.N), and trabecular bone separation (Tb.Sp) to represent the femur character in this part.

### OsO4 Staining and μCT Analysis

We decalcified the long bone in 0.5 M EDTA for 21 days. We cut off the proximal of femurs and then discarded it. The rest of the femur was incubated in aqueous osmium tetroxide (OsO4) (2%, 2 h). Then we rinsed the tissues for 48 h and scanned them by using μCT (9-μm pixel; 45kV, 177 μA). The number and the volume of adipocytes (ad. N; ad. V) in bone marrow were quantified for inter-group analyses in this part.

### Hematoxylin and Eosin and Tartrated Resistant Acid Phosphatase Staining

After 3-weeks decalcification of the femur samples, we embedded them in paraffin and cut them into 4 μm-thick slices for H&E and TRAP staining. The adipocyte number relative to bone marrow area was then collected for inter-group analysis based on the H&E staining; and the osteoclast (OC) numbers were calculated via TRAP staining.

### Immunofluorescence Assay

Harvested femurs were decalcified by 0.5 M EDTA. The tissues were embedded in gelatin (at a concentration of 8%) with sucrose (at a concentration of 20%) in the presence of polyvinylpyrrolidone (at a concentration of 2%). We sliced the tissues into 20 μm-thick sections. Subsequently, we blocked the samples, treated the samples with primary antibodies (the antibodoy for TAZ, 1:200, Abcam; for OCN, 1:200, R&D; for perilipin, 1:400, Sigma), and incubated them with secondary antibodies.

### Cell Isolation and Culture

We euthanized the mice and dissected the bilateral femurs as well as tibias to collect the marrow cells. Then we planted the cells in Dulbecco’s modified Eagle’s medium (DMEM) with fetal bovine serum (FBS, 12%). BMSCs at passage three were incubated with the antibody of CD34, CD45, CD29, and CD90 (Thermo Fisher Scientific, United States) in phycoerythrin and sorted by flow cytometry by different cell fluorescence for identification. To identify the effect of CCG on cell cycles, 3 days treated cells were harvested and fixed with 70% ethanol. After washing in PBS, cells were stained with propidium iodide (Sigma, United States) (5 mg/ml) for 30 min in the dark at 4°C. Fluorescence was measured with the flow cytometer equipped with a 570-nm argon ion laser (Epics XL, Beckman Coulter Corporation, FL) and the data were analyzed using the Muticycle AV software.

### Cell Differentiation

For one thing, we mixed β-glycerophosphoric acid (10 mM, Sigma), dexamethasone (10 nM, Sigma), and ascorbic acid (50 μg/ml, Sigma) with the growth medium to induce osteogenesis. For another thing, we added dexamethasone (1 μM, Sigma), insulin (10 mg/ml, Sigma), and methyl isobutylxanthine (500 mM, Sigma) to the growth medium to induce adipogenesis.

### CCG Administration *In Vitro*

We sub-cultured the cells that reached approximately 80% confluence into a new flask and replaced the growth medium with differential medium in the absence or presence of CCG (10, 100, or 1000 μM). Then, we selected an appropriate concentration of CCG for the next research part.

### Cell Viability Assay

The cells were incubated with MTT (5 mg/ml, Solarbio) to form crystals. We then added dimethyl sulfoxide (DMSO, Solarbio) to fully dissolve the crystals in 10 min. At a wavelength of 490 nm, we measured the absorbance by using the microplate spectrophotometer (BioTek Instruments, United States) to acquire the cell viability assay.

### Plasmid Transfection

We used the plasmid mixed with small interfering RNA sequences for gene knockdown research. The designed and synthesized SiTAZ was used to knock down the TAZ expression (Genechem, China). We used the Lipofectamine 3000 (Thermo Fisher Scientific) for transfection. After 24 h, the transfected cells were ready for the subsequent experiments. Furthermore, we measured the transfection efficiency using flow cytometry (Epics XL, Beckman Coulter Corporation, United States). The sequences of SiTAZ were:

5′-GAT​CCC​CTG​GAC​CAA​GTA​TAT​GAA​CCA​CTC​GAG​TGG​TTC​ATA​TAC​TTG​GTC​CAG​TTT​TTG​GAT-3′;
5′-AGC​TAT​CCA​AAA​ACT​GGA​CCA​AGT​ATA​TGA​ACC​ACT​CGA​GTG​GTT​CAT​ATA​CTT​GGT​CCA​GGG-3′.

### Alizarin Red Staining

We conducted the AR-S after 14-days treatment of osteogenic cocktail. We washed the cells and fixed them with 4% paraformaldehyde. Then we incubated the washed cells with AR (0.1%, Sigma). Then, the cells were de-stained with 10% cetylpyridinium chloride (Sigma) for AR quantification. We detected the OD value at 562 nm wavelength to calculate the calcium concentrations.

### Oil Red O Staining

We conducted the oil red O staining after 14-days treatment of the adipogenic medium. The cells were then incubated with oil red O solution (0.5%, Sigma). After fully washed, we acquired the staining images using the microscope. Then we de-stained the treated cells with isopropanol in PBS for quantification study. We detected the OD value at 520 nm wavelength to calculate the lipid droplets using the microscope (Leica).

### Real-Time Reverse Transcription-Polymerase Chain Reaction

We extracted the total RNA using TRIzol^®^ reagent (Ambition, United States). A total of 1 μg RNA was reversed-transcribed by using cDNA synthesis Kit (Thermo Fisher Scientific). Real-time RT-PCR was performed on a CFX96 Real-Time PCR Detection System (Bio-Rad, Hercules, CA) using SuperReal PreMix Plus (TIANGEN, Beijing, China). All primers were synthesized by Invitrogen (Carlsbad, CA). The relative expression of mRNAs was calculated according to the ratio of the copy numbers of the target genes (TAZ, RUNX2, OCN, PPARγ, perilipin) to the housekeeping gene GAPDH in each sample. The relative gene expression values were evaluated by the 2-△△Ct method [24]. Forward and reverse primers are listed in Supplementary Table S1.

### Western Blotting Analysis

We isolated the proteins from the treated cells seeded in 60-mm plastic dishes. We separated the target proteins using 12% sodium dodecyl sulfate polyacrylamide gel electrophoresis (SDS-PAGE; Costar) and transferred them into a polyvinylidene fluoride (PVDF; Costar) membrane. Membranes were blocked with 5% milk and incubated with primary antibodies against TAZ (1:200; Abcam), RUNX2 (1:200; Cell Signaling), OCN (1:200; Takara Bio), PPARγ (1:100; Cell Signaling), perilipin (1:400; Abcam), p-ERK (1:100; Cell Signaling), ERK (1:100; Cell SignalingA), or GAPDH (1:200, Bioworld, United States). Then we induced the conjugated secondary antibody (IRDye800^®^, 1:2000, Rockland, United States) and scanned the samples using the Odyssey Infrared Imaging System (Li-COR Biosciences, Beijing, China). ImageJ (version 1.46) was used to determine the integrated intensity of the detected band (Tan and Dai, 2019).

### Statistics

Quantitative results were expressed as mean ± standard deviation (SD). *In vivo*, there were five mice in each group; *in vitro*, all experiments were replicated at least three times. Independent samples *t*-test for the comparison of two groups, one-way analysis of variance (ANOVA) followed by Student Newman Keuls (S-N-K) post hoc analysis for the parametric data among multiple groups, which were performed by using SPSS (v.21.0). Values were considered statistically significant at *p* < 0.05.

## Results

### CCG Ameliorated Bone Loss and Reduced Marrow Adiposity of Aging Mice

**FIGURE 1:**
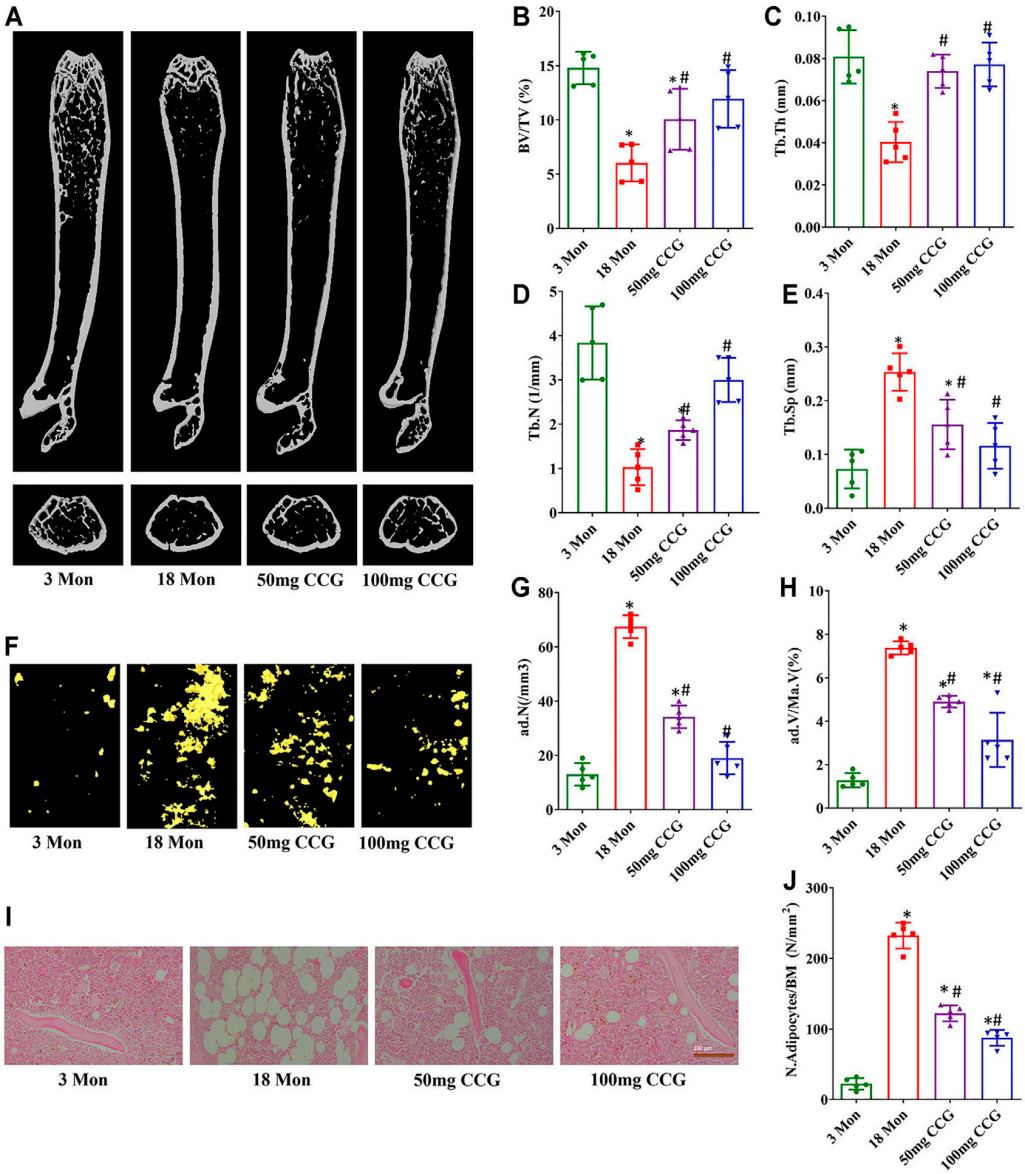
CCG ameliorated bone loss and marrow adiposity of aging mice. **(A–E)** Representative μ-CT images of femurs from the animals in different groups to acquire the quantitative analysis of trabecular bone. **(F–H)** Representative μ-CT images of OsO_4_ staining of femurs from the animals in different groups to acquire the quantitative analysis of the volume of adipocytes. **(I, J)** Representative images of H&E staining to show the ratio of adipocyte number to the area of bone marrow. Scale bar: 100 μm (n = 5) *p < 0.05 vs. the 3-mon mice group; #*p* < 0.05 vs. the 18-mon mice group.

We used μCT to analyze the trabecular bone structure as well as the marrow adipose tissue volume. The BV/TV, Tb.Th, and Tb.N of the femur bone significantly decreased in the 18-mon mice relative to the 3-mon mice, and the Tb. Sp in marrow significantly increased in the 18-mon mice compared with the 3-mon mice (Figures 1A–E). Simultaneously, fat was significantly accumulated with aging, which was demonstrated by more lipid droplets stained with osmium tetroxide (OsO_4_) in the marrow (Figures 1F–H). After oral administration with 50 mg/kg/d or 100 mg/kg/d CCG for 2 months, the age-related bone loss and fat accumulation by aging were markedly reduced. First, the μCT results showed an increased bone formation compared with the 18-mon group (Figures 1A–E). Second, a significantly lower amount of fat droplets appeared in 50 mg/kg/d or 100 mg/kg/d CCG administration group (Figures 1F–H). Similarly, H&E staining calculated a lower number of adipocytes after oral administration of CCG (Figures 1I,J). These data suggested that oral administration of CCG ameliorated bone loss and marrow adiposity of aging mice. Furthermore, CCG was demonstrated to inhibit osteoclasts formation by TRAP staining (Supplementary Figure S1).

### CCG Increased TAZ Expression to Regulate Switch from Adipogenesis to Osteogenesis in Bone Marrow

**FIGURE 2:**
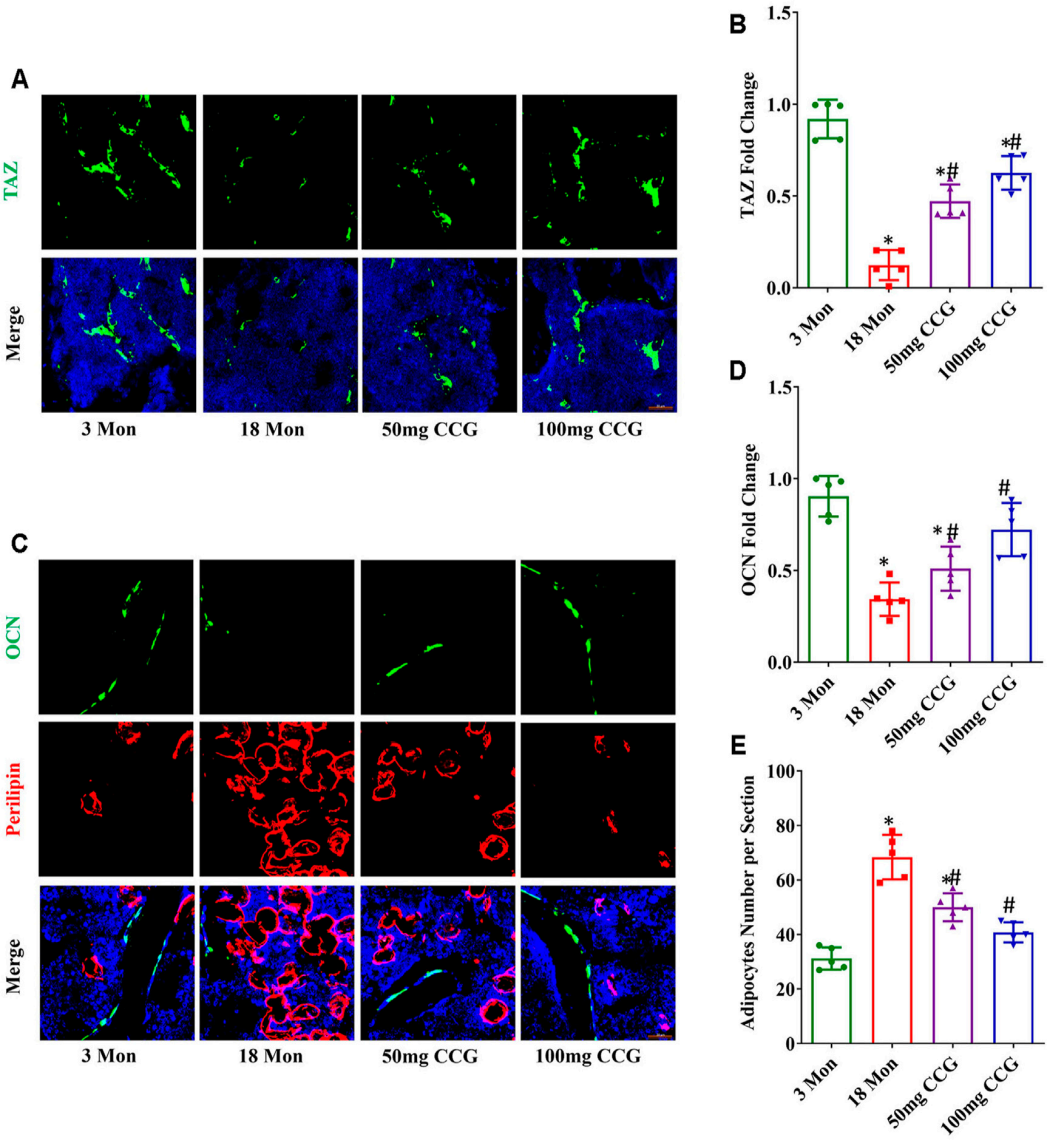
CCG targeted TAZ to regulate the switch from adipogenesis to osteogenesis of BMSCs *in vivo*. **(A, B)** Representative images and quantitative analysis of TAZ (green) expression in the mice femurs from different groups. **(C–E)** Immunofluorescent staining of OCN (green) and perilipin (red) in the mice femurs and the relevant quantitative analysis. Scale bar: 50 μm (n = 5) *p < 0.05 vs. the 3-mon mice group; #*p* < 0.05 vs. the 18-mon mice group.

TAZ expression was decreased with aging, while 50 mg/kg/d or 100 mg/kg/d dose of CCG offset the age-related down-expression of TAZ (Figures 2A,B). Consequently, we found a decreased expression of osteocalcin (OCN) (the marker for osteogenesis) and an increased expression of perilipin (the marker for adipogenesis) in aging mice (Figures 2C–E). CCG administration ameliorated the down-expression of OCN and decreased the up-expression of perilipin in aging mice (Figures 2C–E). Above all, we speculated that CCG targeted TAZ to regulate the switch from adipocytes to osteoblasts of BMSCs. In order to confirm our hypothesis, we conducted experiments *in vitro* study.

### CCG Targeted TAZ to Facilitate Osteogenic Differentiation of BMSCs *In Vitro*

**FIGURE 3:**
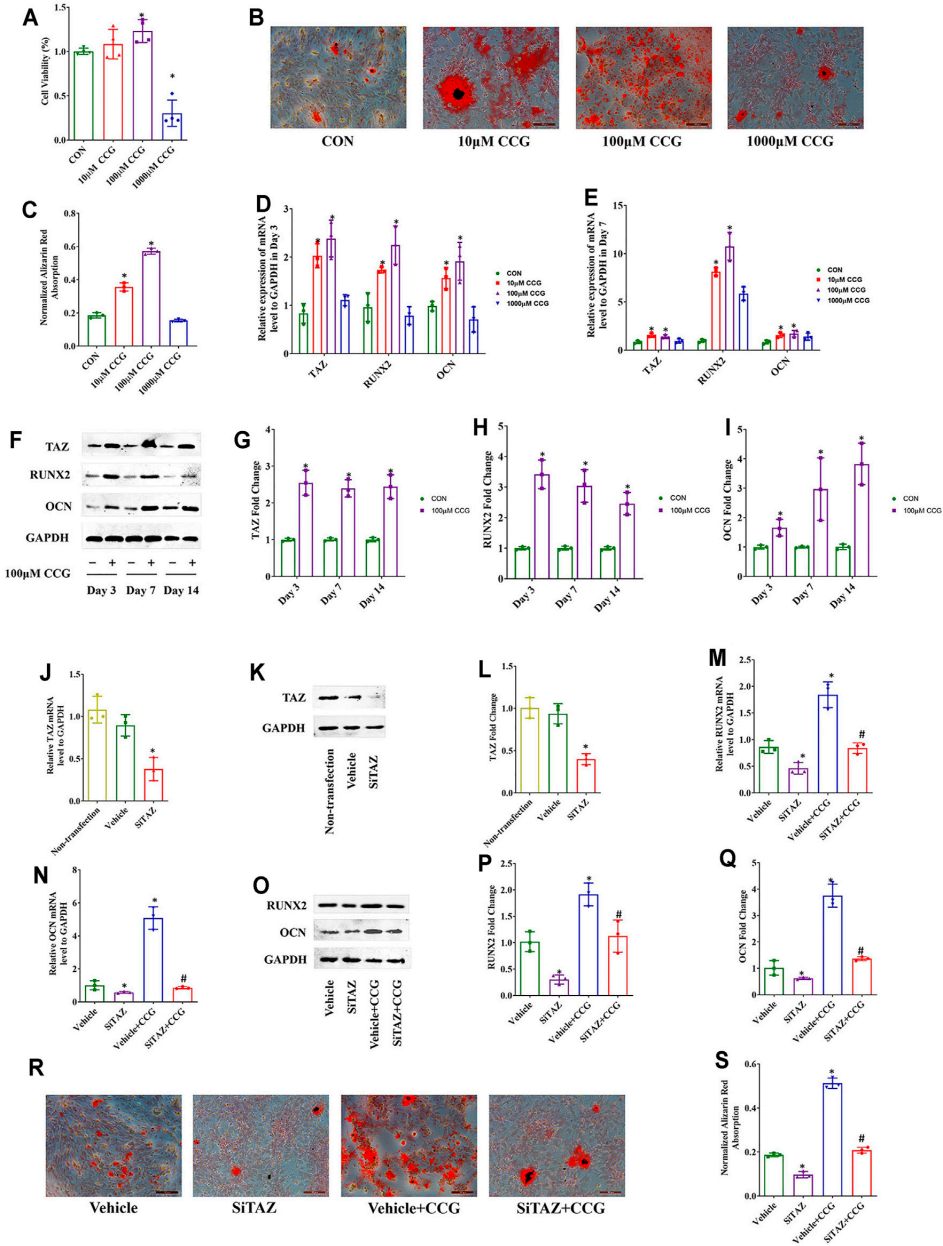
CCG targeted TAZ to facilitate osteogenic differentiation of BMSCs *in vitro*. **(A)** MTT assays presented cell viabilities after CCG administration during osteogenesis of BMSCs. **(B, C)** Representative images of AR-S results in different mice groups. Scale bar: 100 μm. **(D, E)** Relative mRNA levels of TAZ, RUNX2, and OCN with or without CCG treatment. **(F–I)** TAZ, RUNX2, and OCN expression in protein levels with or without CCG treatment. *p < 0.05 vs. the control group. **(J–L)** SiTAZ plasmid significantly knocked down the mRNA and protein levels of TAZ during the osteogenesis of BMSCs. **(M–Q)** SiTAZ discounted the CCG induced up-regulation of RUNX2 and OCN. **(R, S)** Representative images of AR-S showed the differences of calcium deposits in different groups. Bar graphs showed the means ± SD from three independent experiments. Scale bar: 100 μm (n = 3) *p < 0.05 vs. the Vehicle group; #*p* < 0.05 vs. the Vehicle + CCG group.

The hematopoietic surface markers CD34, CD45, CD29, and CD90 were detected to identify the BMSCs (Supplementary Figure S2). Then, we performed MTT assays and found that the cell viabilities were decreased after 1000 μM CCG treatment but increased after administration with 100 μM of CCG. Of interest, 10 μM CCG did not influence the cell viability (Figure 3A). To verify whether the administration of 100 μM CCG influenced the proliferation of BMSCs, we calculated the percentage of the cells in the G1 phase with the flow cytometer after 3 days’ treatment. As a result, we found that the percentage of the cells in the G1 phase did not change after the CCG treatment (Supplementary Figure S3).

In order to confirm whether CCG had anti-aging effects, we identified the expression of p21 and p16 using real-time RT PCR analyses. We found that the CCG treatment induced the down-expression of p21 and p16 (Supplementary Figure S4). Further, CCG was proved to facilitate the most osteogenesis at the concentration of 100 μM. On one hand, 100 μM of CCG accelerated the calcium deposition evidenced by AR-S experiment (Figures 3B,C). On the other hand, the highest mRNA levels of RUNX2 and OCN expressed in 100 μM CCG group (Figures 3D,E). Thus, 100 μM CCG was administrated on cells in the subsequent experiments. Inevitably, 100 μM of CCG markedly increased the expression of TAZ, RUNX2, and OCN on Day 3, 7, and 14 (Figures 3F–I). Meanwhile, the adipogenic markers were down-regulated during osteogenesis by CCG treatment (Supplementary Figure S5A). Then, the TAZ expression was knocked down by transfecting with plasmids containing SiTAZ sequences (Figures 3J–L). SiTAZ significantly offset the CCG induced up-expression of the markers for osteogenesis, RUNX2, and OCN (Figures 3M–Q). Moreover, the AR-S results suggested SiTAZ delayed the recruitment of osteogenic nodules by CCG (Figures 3R, S). Above all, CCG might facilitate osteogenesis by targeting TAZ with a peak at the concentration of 100 μM for BMSCs differentiation.

### CCG Targeted TAZ to Reduce Adipogenic Differentiation of BMSCs *In Vitro*

**FIGURE 4:**
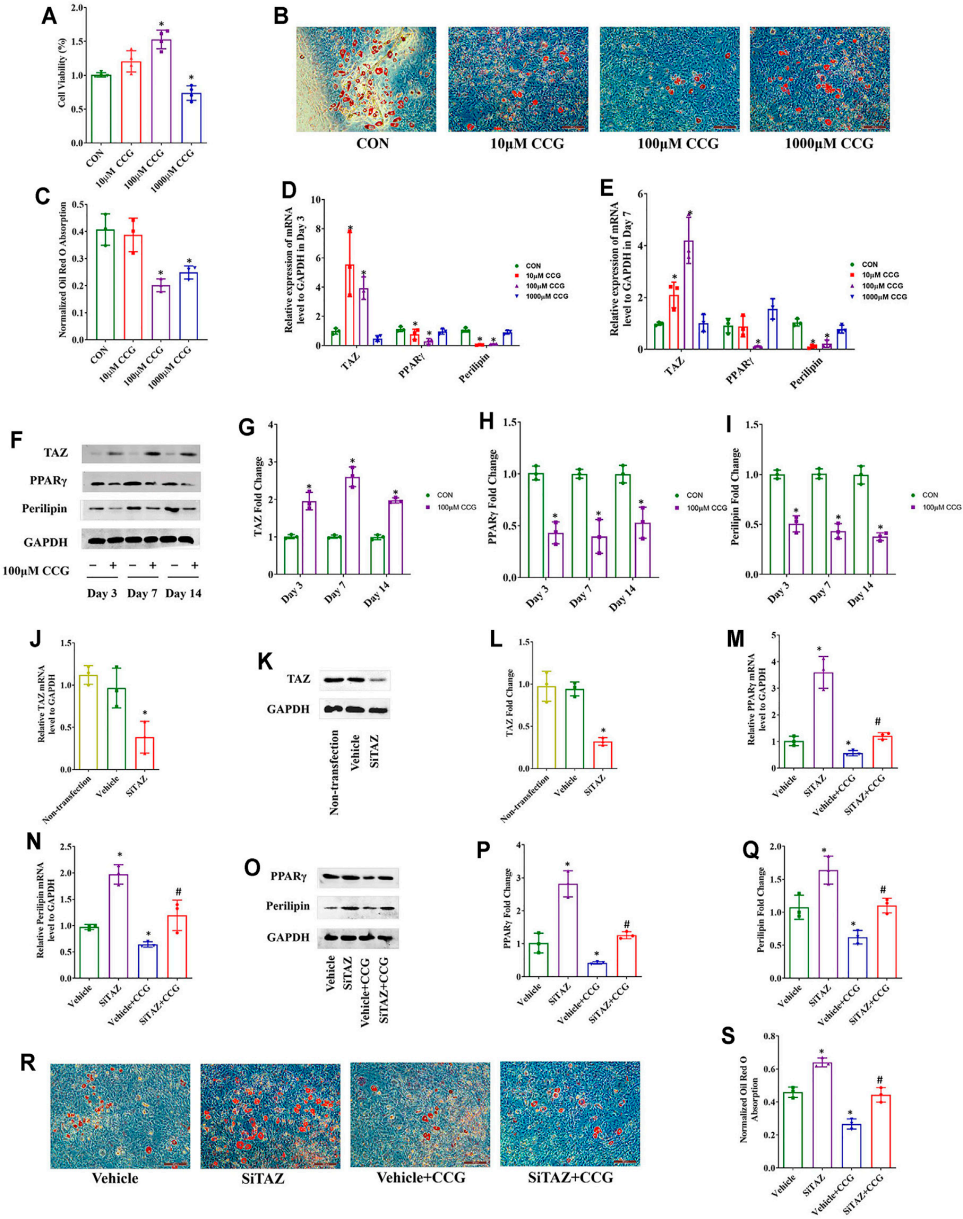
CCG targeted TAZ to reduce adipogenic differentiation of BMSCs *in vitro*. **(A)** MTT assays presented cell viabilities after CCG administration during adipogenesis of BMSCs. **(B, C)** Representative images of Oil Red O staining reflected the lipid droplets. Scale bar: 100 μm. **(D, E)** Relative mRNA levels of TAZ, PPARγ, and perilipin to GAPDH were presented in Day 3 and Day 7 in the absence or presence of CCG during adipogenesis. **(F–I)** Relative protein levels of TAZ, PPARγ, and perilipin to GAPDH were presented in Day 3, in Day 7, and in Day 14 after the treatment. *p < 0.05 vs. the control group. **(J–L)** Relative mRNA and protein levels to GAPDH of TAZ were significantly knocked down by the SiTAZ plasmid during adipogenesis. **(M–Q)** SiTAZ discounted the CCG induced up-expression of TAZ and down-expression of PPARγ and perilipin at Day 3 after the treatment. **(R, S)** Representative images of Oil Red O staining showed the lipid droplets in different groups. Bar graphs showed the means ± SD from three independent experiments. Scale bar: 100 μm (n = 3) *p < 0.05 vs. the Vehicle group; #*p* < 0.05 vs. the Vehicle + CCG group.

As well, we performed MTT assays and found that the cell viabilities were decreased after 1000 μM CCG administration but increased after administration with 100 μM of CCG during adipogenic differentiation of BMSCs, and 10 μM CCG did not influence the cell viability (Figure 4A). The Oil Red O staining results demonsrated that 100 μM of CCG decreased the lipid droplets during adipogenesis on Day 14 (Figures 4B,C). Consistently, the lowest mRNA levels of PPARγ and perilipin but the highest TAZ expression appeared in 100 μM CCG group during adipogenesis (Figures 4D,E). Meanwhile, the osteogenic markers were up-regulated during the administration of CCG treatment (Supplementary Figure S5B). Thus, 100 μM CCG was also administrated on cells in the subsequent experiments during adipogenesis. Inevitably, the expression of PPARγ and perilipin was markedly decreased and the TAZ protein levels were significantly increased by 100 μM CCG administration during adipogenesis (Figures 4F–I). Then the TAZ expression was knocked down by transfecting with plasmids containing SiTAZ sequences (Figures 4J–L). Both at the mRNA and protein levels, TAZ knockdown markedly lessened the CCG induced down-expression of PPARγ and perilipin (Figures 4M–Q). Accordingly, the Oil Red O staining also points a link between CCG treatment and TAZ signaling transduction during adipogenesis of BMSCs (Figures 4R, S). Taken together, we hypothesized that CCG targeted TAZ to reduce marrow adiposity *in vitro*.

### CCG Activated p-ERK Signal to Modulate TAZ Expression and BMSCs Lineage Commitment

**FIGURE 5:**
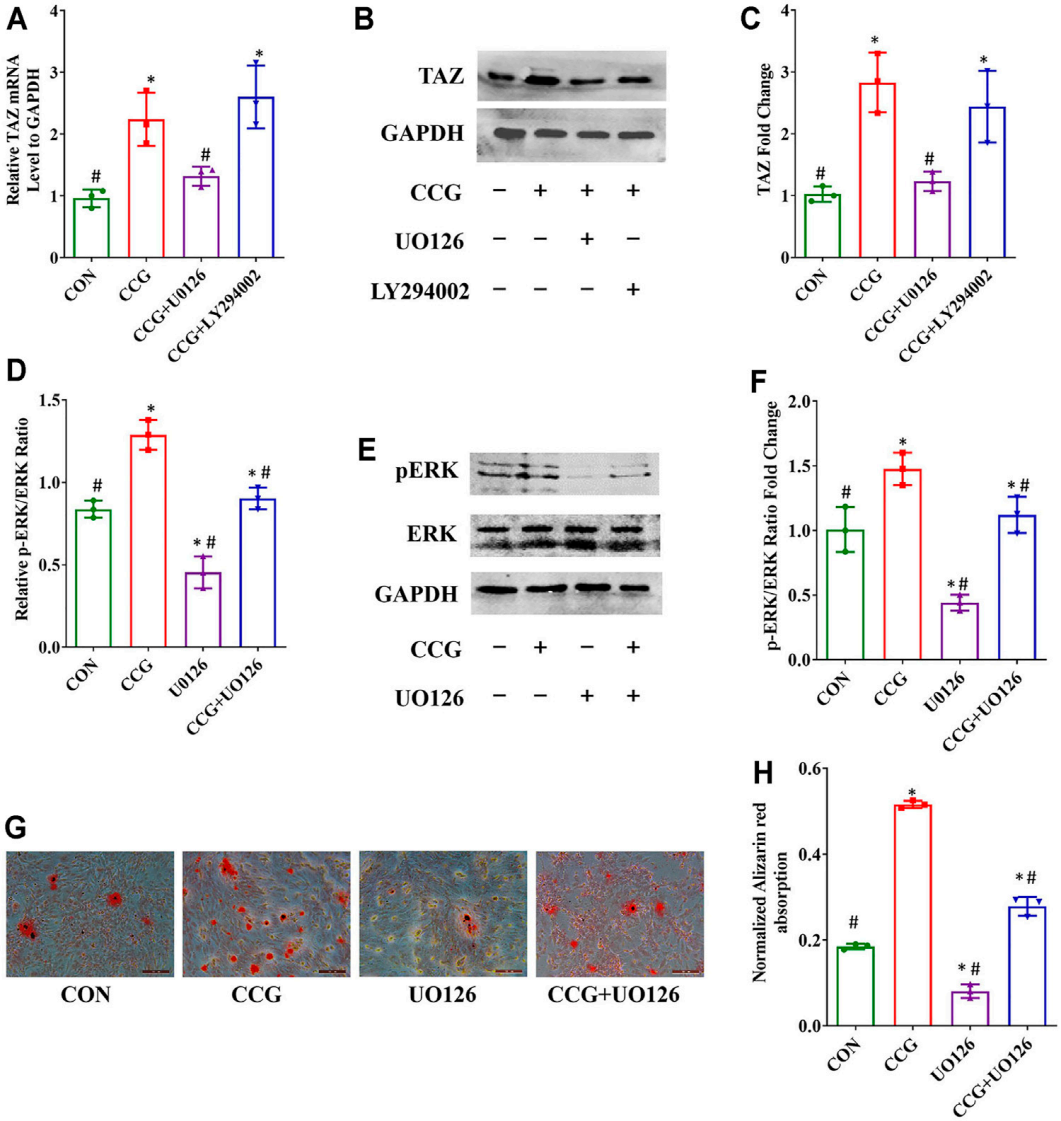
Osteogenic differentiation and TAZ expression were increased by CCG treatment by increasing the p-ERK/ERK ratio. **(A–C)** Relative mRNA and protein levels to GAPDH of TAZ at Day 3 in different groups. **(D–F)** Relative mRNA and protein levels of p-ERK at Day 3 in different groups. **(G, H)** Representative images of AR-S showed the calcium deposits were influenced by CCG treatment. Scale bar: 100 μm *p < 0.05 vs. the control group (cells cultured in osteogenic medium during osteogenesis); #*p* < 0.05 vs. the CCG administration group.

**FIGURE 6:**
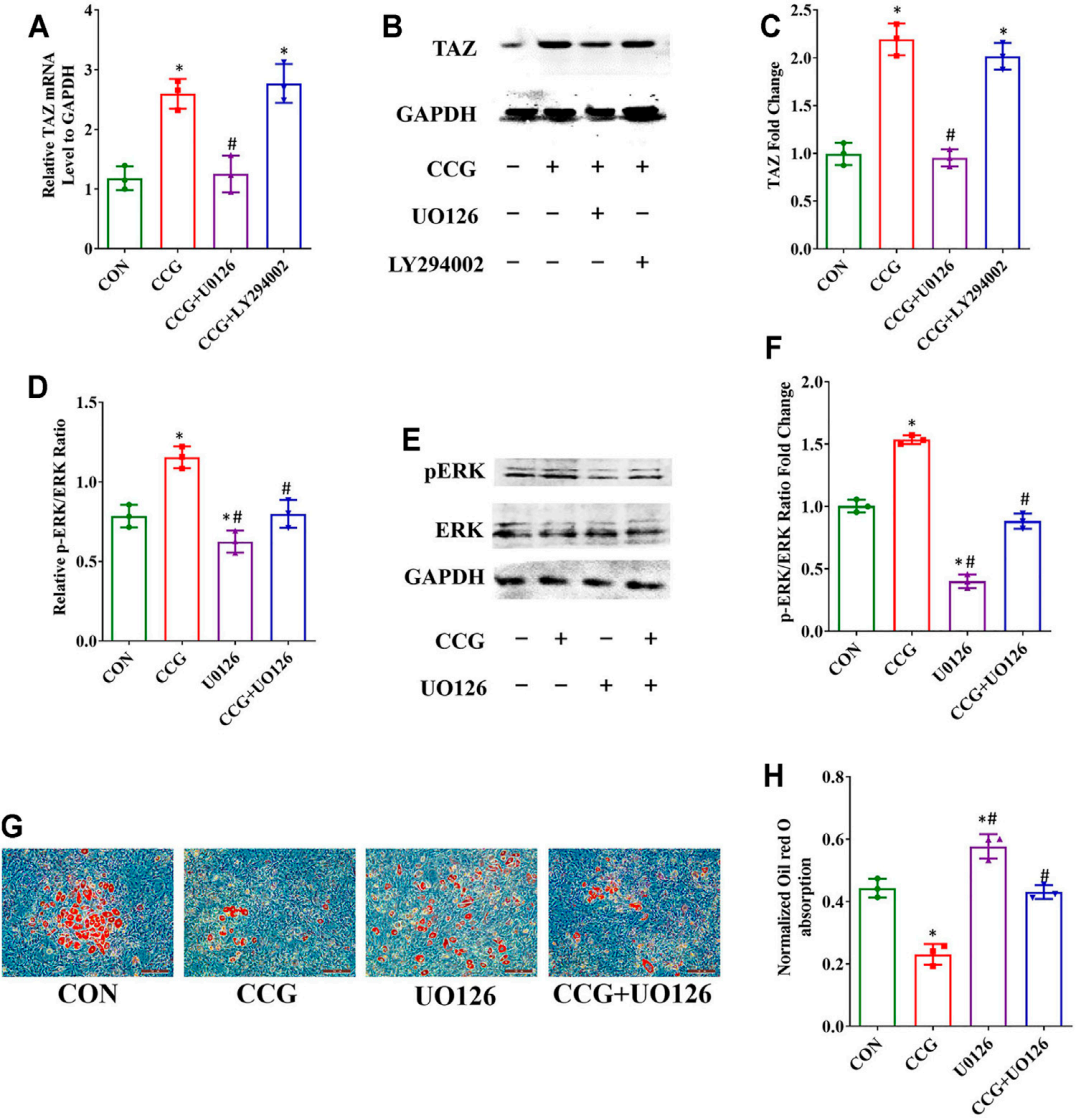
Decreased adipogenic differentiation accompanied by increased TAZ expression was also mediated by p-ERK/ERK signaling after CCG treatment. **(A–C)** Relative mRNA and protein levels of TAZ to GAPDH at Day 3 after the treatment during adipogenesis. **(D–F)** Relative mRNA and protein levels of p-ERK at Day 3 after the treatment during adipogenesis. **(G, H)** Representative images of Oil Red O staining showed the lipid droplets were lessened by CCG. Scale bar: 100 μm (n = 3) *p < 0.05 vs. the control group (cells cultured in adipogenic medium during adipogenesis); #*p* < 0.05 vs. the CCG treatment group.

To elucidate the signaling transduntion, we conducted inhibitor study using pathway inhibitors. We found that the up-expression of TAZ induced by CCG administration was significantly diminished by UO126, rather than LY294002 (Figures 5A–C). Furthermore, the p-ERK/ERK ratio could be augmented by CCG, suggesting CCG could stimulate the MEK-ERK signaling transduction during osteogenesis (Figures 5D–F). Additionally, the AR-S results reflected that UO126 attenuated the formation of osteogenic nodules accelerated by CCG (Figures 5G,H). Similar results were confirmed during adipogenesis to that during osteogenesis (Figure 6). We concluded that the MEK-ERK signaling pathway was involved in the CCG-TAZ axis to facilitate osteogenesis at the expense of adiposity in bone marrow for anti-aging related osteoporosis.

## Discussion

Osteoporosis is recognized as a common concern in the aging population worldwide (Curtis et al., 2015). Osteogenesis reduction and fat accumulation triggered an imbalance of bone remodeling (Infante and Rodríguez, 2018). With aging progressing, senile osteoporosis becomes a growing public problem. The importance of developing new anti-osteoporosis therapies targeted at osteoblasts not only to increase bone formation and induce bone growth, but also to prevent age-related fat accumulation (Li et al., 2015). Increasing evidences bestow Chinese herbs vital roles in the therapy of osteoporosis and bone fracture, with Curculigo orchioides identified as widely used in senile osteoporosis (Wang et al., 2012; Wu et al., 2012; Tan et al., 2019). The potential biological functions and molecular mechanisms of the effects of CCG, the major bioactive component of Curculigo orchioides, on the switch from adipocytes into osteoblasts in aging mice has never been reported.

In our present study, the μCT analyses revealed that oral administration of CCG ameliorated the bone loss and marrow adiposity in the bone of aging mice. Previously, researchers reported that CCG prevented bone loss in ovariectomized rats (Shen et al., 2013). Consistently, other researchers have demonstrated that CCG stimulated the secretion of bone morphogenetic protein 2 (BMP-2) to promote osteogenesis (Ma et al., 2011). As was reported, CCG also prevented oxidative damage and inhibited osteoclastogenesis in rat bone marrow cells (Wang et al., 2012). As well, our study provided new insight into the molecular mechanisms how CCG regulates the age-related adipocytes-osteoblasts lineage commitment through MEK/ERK-TAZ axis. Thus, CCG might be a good candidate for further development as an anti-osteoporotic remedy in an aging population.

Latent bone-lining osteoblast-adipocyte precursors, including BMSCs, are activated in response to various stimuli including exogenous and endogenous changes causing pre-osteoblasts to increase surface expression of osteogenic markers at the expense of reduced adipogenic markers (Paspaliaris and Kolios, 2019). In addition, the microenvironment significantly affects bone mass by the regulation of vital factors (Du et al., 2019). For instance, TAZ exerted pivotal effects on stem cell fate determination (Panciera et al., 2017; Salem and Hansen, 2019; Zheng and Pan, 2019). In our previous study, we confirmed TAZ as an important transcriptional modulator during osteoblastogenesis, which was evoked by insulin-like growth factor 1 (IGF-1) (Xue et al., 2013). In the present study, we confirmed that the TAZ signaling was involved in the regulation of the differential shift from adipogenesis to osteogenesis by CCG. *In vivo*, the down-expression of TAZ in aging mice was ameliorated by the oral administration of CCG with up-expression of osteogenic genes and down-expression of adipogenic genes. *In vitro*, TAZ knockdown could offset CCG-regulated expression of osteogenic genes and adipogenic genes. Hereby, our results suggested that CCG influenced the age-related switch of cells lineage commitment of BMSCs via TAZ signal pathway.

As recognized commonly, mitogen activated protein kinases (MAPKs)-mediated signaling pathways pronounced markedly in cell differentiation and proliferation (Elango et al., 2019; Kim et al., 2019). Differential molecular in signaling pathways goes through the plasma membrane from extracellular to intracellular environment, then regulates the osteogenesis and adipogenesis (Husain and Jeffries, 2017; Wu et al., 2017; Bukowska et al., 2018). In the early 1990s, as one of the members of the MAPK family, the MEK-ERK pathway was first recognized (Xu et al., 2016). The activation of p-ERK had been shown to be essential for RUNX2 up-regulation and transcriptional activity previously (Li et al., 2017). As was proved, TAZ directly combined to RUNX2 to promote osteogenesis (Wang et al., 2019b). Previously, our study also pointed a molecular link of MEK-ERK pathway to the TAZ during BMSCs osteogenesis (Xue et al., 2013; Wang et al., 2019a; Tan and Dai, 2019). Here, we found that CCG up-regulated TAZ signaling via the MEK-ERK pathway, which might be identified as the underlying regulation network. The MEK-ERK pathway was activated upon CCG-mediated osteogenic differentiation of BMSCs, while the MEK-ERK inhibitors, UO126 reduced the CCG-mediated calcium deposits during cell differentiation. These findings suggested that MRK-ERK activation was the upstream signal by which CCG-induced TAZ up-expression and then regulated the cell differentiation.

## Conclusion

**FIGURE 7:**
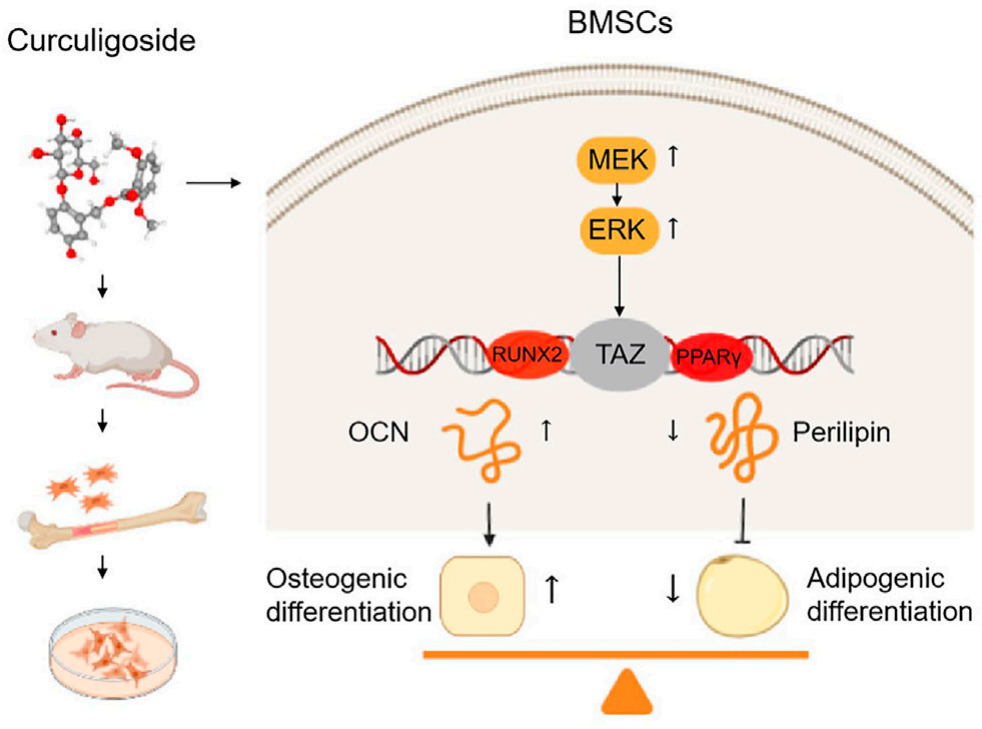
Curculigoside ameliorates bone loss by influencing mesenchymal stem cell fate in aging mice. CCG ameliorated age-related bone loss by upregulating TAZ expression to induce switch from adipogenesis to osteogenesis of BMSCs in aging mice through MEK/ERK-TAZ axis.

Our present study confirmed that CCG ameliorated age-related bone loss by upregulating TAZ expression to induce the switch from adipogenesis to osteogenesis of BMSCs in aging mice through MEK/ERK-TAZ axis. *In vivo*, oral administration of CCG significantly increased the bone mass and decreased the adipocyte numbers in the bone marrow of aging mice. *In vitro*, a proper concentration of CCG regulated TAZ expression through the MEK-ERK pathway to facilitated osteogenesis at the expense of reduced adipogenesis of BMSCs (Figure 7).

All these results revealed that CCG targeted TAZ to ameliorate bone loss in aging mice, which provided the evidence for the clinical use in age-related osteoporosis. In summary, our study pointed the new link of CCG to TAZ signaling and provided a novel therapeutic target for senile osteoporosis.

## Data Availability

The raw data supporting the conclusions of this article will be made available by the authors, without undue reservation.
