# Supplementary material for: Curculigoside Ameliorates Bone Loss by Influencing Mesenchymal Stem Cell Fate in Aging Mice
Source: Front Cell Dev Biol. 2021 Dec 3;9:767006. doi: 10.3389/fcell.2021.767006 (PMC8678408; doi:10.3389/fcell.2021.767006)
Supplement: Supplementary file 1 [file DataSheet1.docx]

Supplementary Material

**Supplementary figure 1**

**
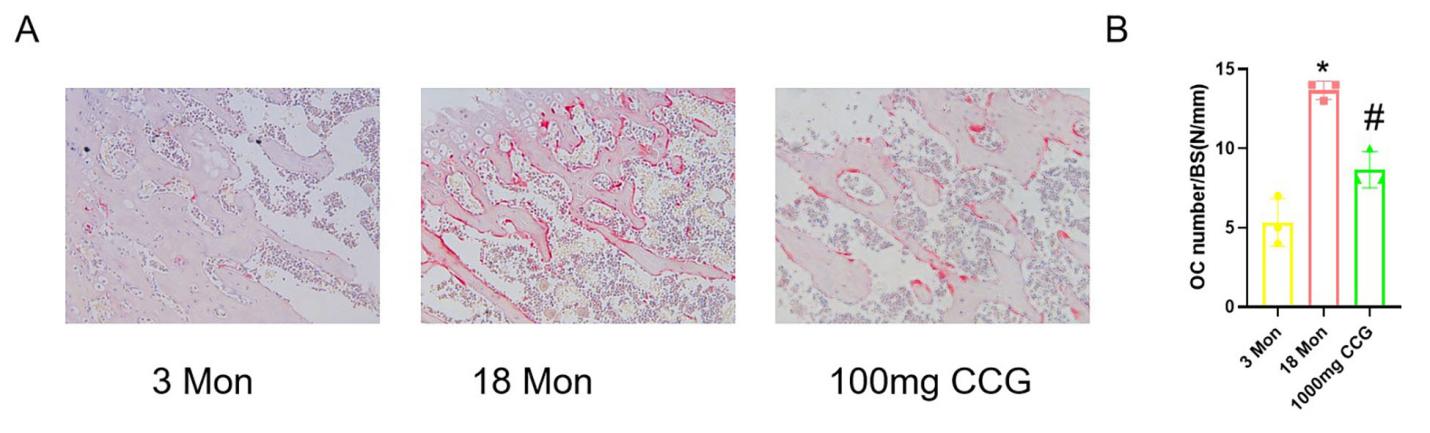
**

Supplementary figure 1: CCG treatment inhibited osteoclast formation. (A) TRAP staining results in different groups. (B) The quantitative analysis for the TRAP staining results. *P < 0.05 vs the 3Mon group; #P < 0.05 vs the 18Mon group.

**Supplementary figure 2**

**
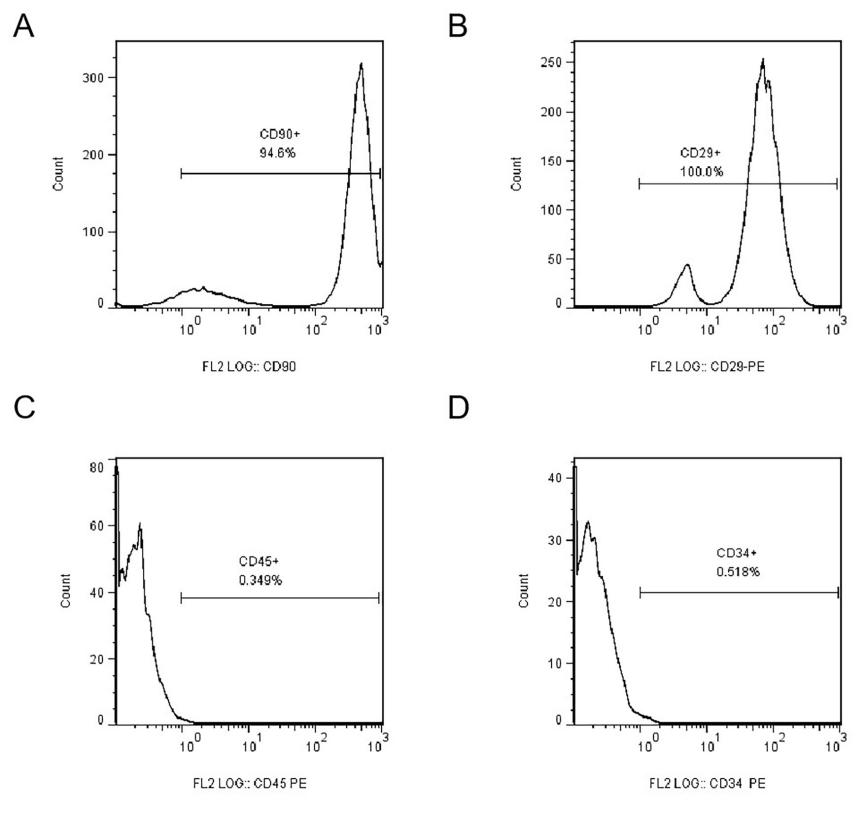
**

Supplementary figure 2: The identification of BMSCs. BMSCs were identified with the positive markers of CD90 and CD29, and the negative markers of CD45 and CD34.

**Supplementary figure 3**

**
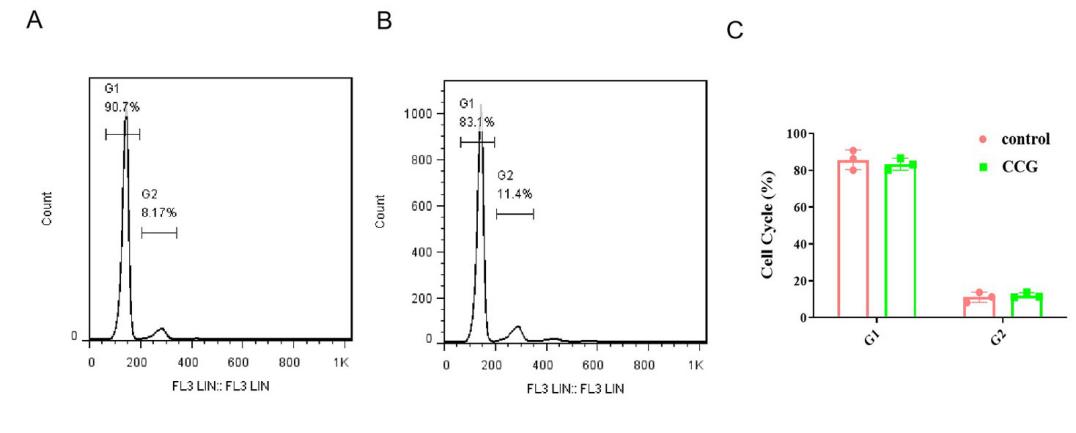
**

Supplementary figure 3: The effects of CCG on the proliferation of BMSCs. (A, B) The percentages of cells in G1, S and G2 phases were shown in the control or CCG treatment group. (C) Quantification analysis of the flow cytometry results. *P < 0.05 vs the control group.

**Supplementary figure 4**

**
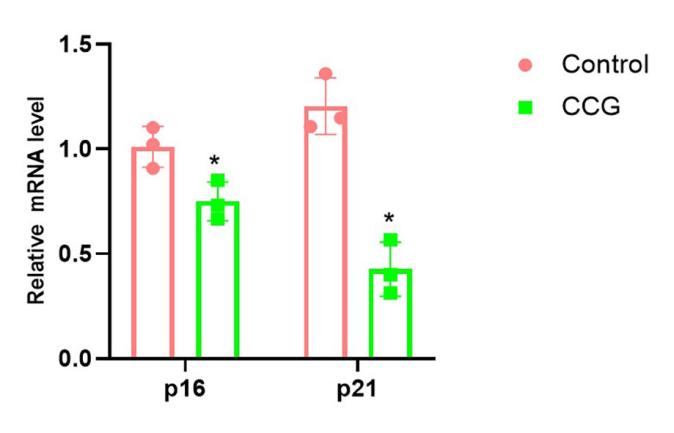
**

Supplementary figure 4: The relative expression of p16 and p21. *P < 0.05 vs the control group.

**Supplementary figure 5**

**
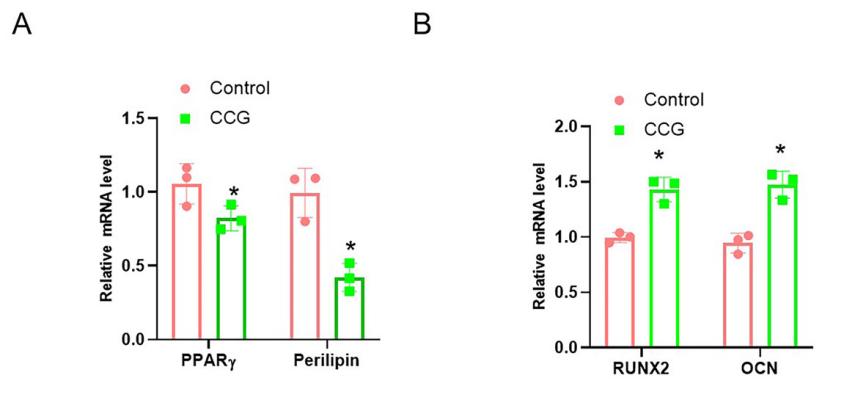
**

Supplementary figure 5: CCG influence the adipogenic markers during osteogenesis as well as the osteogenic markers during adipogenesis. (A) The expression of adipogenic markers was down-regulated by CCG treatment during osteogenesis. (B) The expression of osteogenic markers was up-regulated by CCG treatment during adipogenesis. *P < 0.05 vs the control group.

Supplementary table 1: Forward and reverse primers for real-time RT-PCR

| Primers | Forward | Reverse |
| --- | --- | --- |
| GAPDH | 5’-GCAAGTTCAACGGCACAG-3’ | 5’-CGCCAGTAGACTCCACGAC-3’ |
| TAZ | 5’-GTCACCAACAGTAGCTCAGATC-3’ | 5’-AGTGATTACAGCCAGGTTAGAAAG-3’ |
| RUNX2 | 5’-GGACTGGGTATGGTTTGTAT-3’ | 5’-GCTGAAGAGGCTGTTTGA-3’; |
| OCN | 5’-ACCACATCGGCTTTCAGG-3’ | 5’-CATAGGGCTGGGAGGTCA-3’ |
| PPARγ | 5’-CCTTGCTGTGGGGATGTCTCA -3’ | 5’-CTCCTTCTCGGCCTGTGGCAT -3’; |
| Perilipin | 5’-AGAGTTCTGCAGCTGCCTGTG-3’ | 5’-CAGAGGTGCTTGCAATGGGCA-3’ |
| p16 | 5’-CTTCCTGGACACGCTGGTG-3’ | 5’-ATGGTTACTGCCTCTGGTGC-3’ |
| P21 | 5’-GCGACTGTGATGCGCTAATG-3’ | 5’-GAAGGTAGAGCTTGGGCAGG-3’ |
